# Inferring co-expression networks of *Arabidopsis thaliana* genes during their interaction with *Trichoderma* spp.

**DOI:** 10.1038/s41598-023-48332-w

**Published:** 2024-01-30

**Authors:** Javier-David Vega-Arroy, Alfredo Herrera-Estrella, Cesaré Ovando-Vázquez, Sergio Casas-Flores

**Affiliations:** 1 IPICYT, División de Biología Molecular, Laboratorio de Genómica Funcional y Comparativa, Camino a la Presa San José 2055. Col. Lomas 4 Sección, 78216 San Luis Potosí, SLP Mexico; 2 Centro de Investigación y de Estudios Avanzados del IPN, unidad de Genómica Avanzada-Langebio, Libramiento Norte carretera Irapuato-León km 9.6, 36824 Irapuato, GTO Mexico; 3 IPICYT, CONAHCYT, Centro Nacional de Supercomputo, Laboratorio de Inteligencia Artificial y Bioinformática, Camino a la Presa San José 2055. Col. Lomas 4 sección, 78216 San Luis Potosí, SLP Mexico

**Keywords:** Computational biology and bioinformatics, Gene ontology, High-throughput screening

## Abstract

Fungi of the *Trichoderma* genus are called "biostimulants" because they promote plant growth and development and induce disease resistance. We used conventional transcriptome and gene co-expression analyses to understand the molecular response of the plant *Arabidopsis thaliana* to inoculation with *Trichoderma atroviride* or *Trichoderma virens*. The transcriptional landscape of the plant during the interaction with these fungi showed a reduction in functions such as reactive oxygen species production, defense mechanisms against pathogens, and hormone signaling. *T. virens*, as opposed to *T. atroviride*, was more effective at downregulating genes related to terpenoid metabolism, root development, and chemical homeostasis. Through gene co-expression analysis, we found functional gene modules that closely link plant defense with hypoxia. Notably, we found a transcription factor (locus AT2G47520) with two functional domains of interest: a DNA-binding domain and an N-terminal cysteine needed for protein stability under hypoxia. We hypothesize that the transcription factor can bind to the promoter sequence of the GCC-box that is connected to pathogenesis by positioned weight matrix analysis.

## Introduction

Plants are naturally exposed to abiotic and biotic stresses that affect their growth and reproduction. Abiotic stress is triggered by physical factors such as temperature, drought, and salinity, among others. In contrast, biotic stress is caused by living organisms (or biological entities), including insects, bacteria, fungi, oomycetes, viruses, or other plants^1^.

Stress triggers different responses in plants, such as reprogramming of gene expression, changes in cell metabolism, and alterations in the vegetative and reproductive phases^1^. Biotic stress directly deprives the plant of its nutrients, provokes tissue damage, and could lead to death^1^. Biotic interactions, however, are not always unfavorable to plants since they can also be established with beneficial organisms. Plant-beneficial microorganisms comprise specific groups of bacteria and fungi that have positive effects, such as plant growth promotion, increases in nutrient uptake, and might even activate defense mechanisms. Conversely, pathogenic organisms provoke plant disease and may cause tissue damage or death^2^. In recent decades, research has focused on studying plant–microbe beneficial mutualistic interactions due to their implications in agriculture^3^.

Plants have evolved various defense mechanisms against attack by pathogens. The first layers of plant defense against pathogens comprise the physical and chemical mechanisms. Subsequently, an innate immune response may be established. Basal resistance is a type of local resistance that involves the detection of pathogen-associated molecular patterns (PAMPs) by pattern recognition receptors (PRR), leading to PAMP-triggered immunity (PTI)^4^. In response to plant hostility, pathogens counteract with effector molecules to block the plant defense signal transduction, triggering effector susceptibility (ETS). To hinder pathogen effectors activity, plants evolved resistance proteins (R) of the NLR (nucleotide-binding/leucine-leucine-rich-repeat) family, commonly found in the cytoplasm. NLRs directly or indirectly recognize their cognate effectors, leading to effector-triggered immunity (ETI)^5–7^.

Furthermore, PAMPs and effectors can trigger the systemic acquired resistance (SAR) at distant parts of the original point of infection, mediated by the phytohormone salicylic acid (SA). *Arabidopsis* mutants defective in SA biosynthesis or responsiveness are compromised in basal defense and SAR^8^. SAR is effective against biotrophic and hemibiotrophic microorganisms^9^. In addition, PAMPs or MAMPs (microbe-associated molecular patterns) of necrotrophic or beneficial microorganisms can trigger induced systemic resistance (ISR) through the phytohormones jasmonate/ethylene (JA/ET) pathways^10^. Intriguingly, the interaction of plants with mutualistic microorganisms result in plant growth stimulation, biomass gaining, and changes in root architecture that lead to a more efficient nutrient uptake^11^.

Species of the *Trichoderma* genus are cosmopolitan filamentous fungi of agricultural interest, since several isolates have been used as biological control agents of phytopathogens because they feed on other fungi, produce antibiotics, and are rhizosphere competent^11^. Furthermore, *Trichoderma* spp. colonize the plant root surface to establish mutualistic relationships with plants, modifying root architecture^12^, increasing nutrient uptake capacity and growth^13^. In addition, *Trichoderma* simultaneously induces SAR and ISR to render plants resistant to a wide range of pathogens with different lifestyle^12,14,15^.

In addition, after their interaction with *Trichoderma*, plants become more tolerant to abiotic stress. Mona et al*.*^16^ reported increased drought resistance of tomato plants inoculated with *Trichoderma harzianum.* According to these authors, *Trichoderma* mitigates the harmful effects of drought in the host, which correlates with an increase in secondary metabolites such as phenols, flavonoids, indole acetic acid (IAA), indole butyric acid and gibberellic acid*.* Similarly, Zhang et al.^17^ reported that cucumber plants (*Cucumis sativus*) treated with *T. atroviride* HN082102.1 show increased tolerance to high concentration of NaCl (sodium chloride).

It has been proposed that low oxygen concentrations (hypoxia) could trigger resistance in plants against pathogens, thus suggesting a cross-talk between hypoxia and plant immunity^18^. Like animals, higher plants are aerobic organisms because oxygen is essential for their metabolism and growth. In nature, plants experience hypoxia under excessive rainfall or flooded soils, resulting in low oxygen concentration in the rhizosphere^19^. This hypoxia leads plants to fine-tune growth and metabolism to adapt under such conditions. Ethylene response transcription factors (ERFs) are known to play an important role in hypoxia. Some rice varieties have adapted to hypoxia thanks to transcription factors known as "snorkels" that respond to ethylene, allowing them to adapt their growth ^20^. In *A. thaliana*, oxygen regulates the stability of the ERF-VII family of transcription factors. These proteins have an N-terminal cysteine targets them for degradation by the proteasome in the presence of oxygen. On the contrary, in the absence of oxygen, the ERF-VII is stabilized and translocated to the nucleus, where they induce the transcription of genes to alleviate hypoxia ^2^.

Although the *Trichoderma*-plant interaction is a well-studied phenomenon, we need more information to elucidate the molecular mechanisms that govern the functional associations between biological processes in plants during their interaction with the fungus. Conventional approaches that usually characterize one or two genes do not represent the complex adaptation mechanism of the plant against biotic and abiotic stresses. However, with the advent of “omic sciences" in combination with bioinformatics, various mathematical methodologies have been proposed to analyze massive amounts of data that allow us to understand these biological interactions. In this regard, in transcriptomic analysis of tomato interacting with *T. harzianum* (T22) and aphids, the induction of early and late defense genes clarified the dynamics in the behavior of genes in time^21^. They identified genes with an indirect role in tomato defense against insects, such as sesquiterpene synthase and geranylgeranyl phosphate synthase encoding genes. de Palma et al.^22^, based on transcriptomic analysis, proposed that the promotion of growth in tomato plants by *T. harzianum* was due to the differential expression of nutrient transporter genes and the modifications in root architecture through the ethylene/indole-3-acetic acid signaling pathways.

The analysis of co-expression networks allows us to simultaneously identify and explore thousands of genes with similar expression patterns under certain conditions that are grouped to respond to a stimulus. This methodology aims to identify causal variables and regulatory mechanisms that control these processes^23^.

Gene co-expression network analysis is a mathematical methodology that helps to explore and visualize how genes are associated in a biological system to fulfill a specific function. Co-expression network analysis has its theory behind it, known as the graph theory. This theory is a mathematical methodology whose origin dates to 1736 and has been successfully applied in biological sciences to analyze phenomena such as protein interactions, protein structural analysis, gene expression, and microbiome phylogeny^23–26^.

We can generically define a network as a set of actors (nodes) maintaining certain connections between them (edges)^27^. Network analysis makes it easier for us to identify central nodes. These central nodes represent genes that are master regulators in a network of genes and typically play essential roles in a biological system^27^.

Here, the transcriptional response of the model plant *Arabidopsis thaliana* during its interaction with *Trichoderma virens* or *Trichoderma atroviride* was analyzed. We performed a gene co-expression network analysis. These analyses were assessed from a mathematical perspective based on their centrality measures to identify functional modules and relevant genes during the plant-fungus interaction.

## Results

### Differential expression analysis

**Figure 1:**
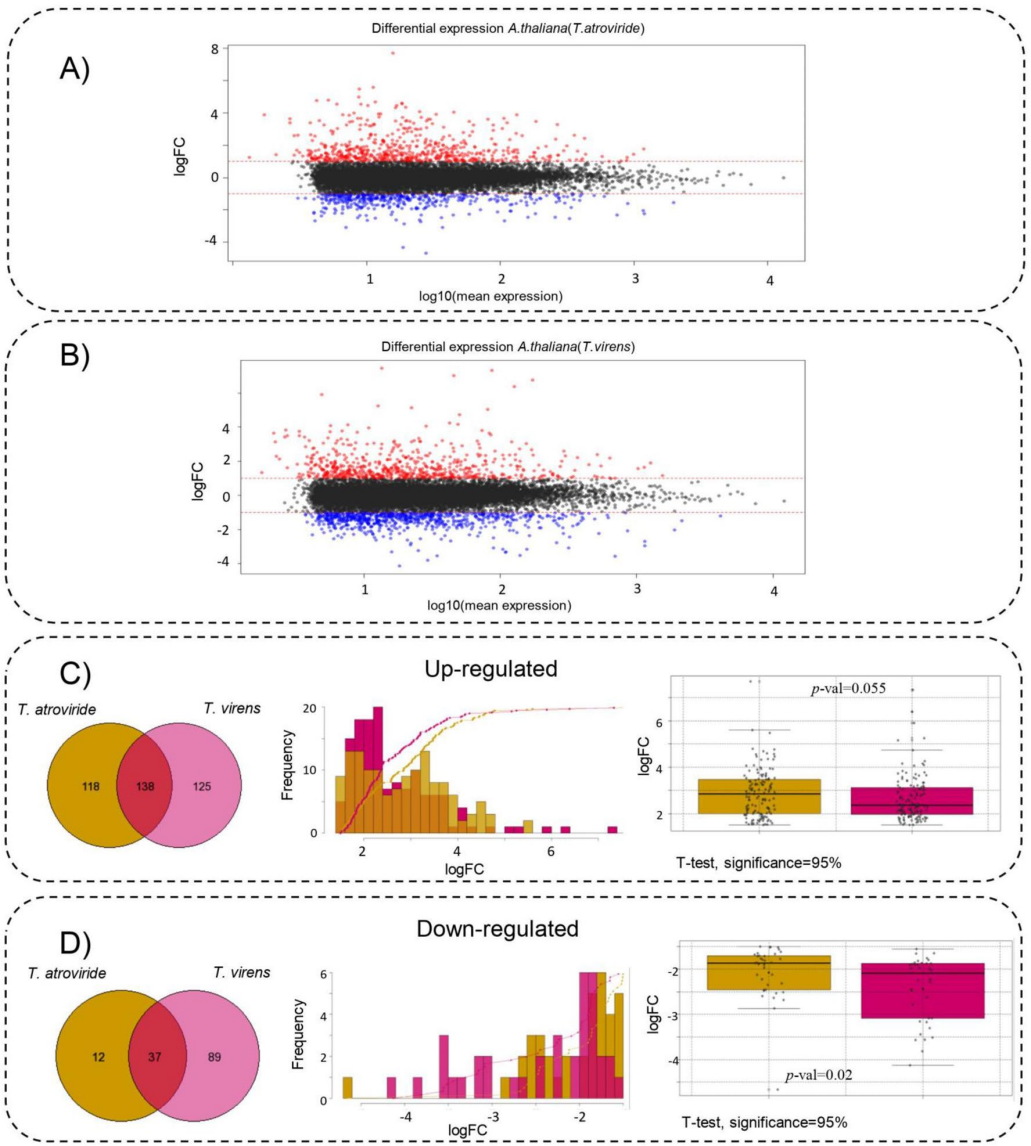
Differential expression analysis. (**A**) MA-plot of the differential expression analysis *A.thaliana*_*(T.atroviride)*_. On y-axis is show the log_2_ fold changes versus x-axis represents log_10_ the average expression signal. Red dots represent up-regulated genes; blue dots represent down-regulated genes. (**B**) MA-plot of the differential expression analysis *A.thaliana*_*(T.virens)*_. On y-axis is show the log_2_ fold changes versus x-axis represents log_10_ the average expression signal Red dots represent up-regulated genes; blue dots represent down-regulated genes. (**C**) Comparison of genes up-regulated in *A. thaliana* by *T. atroviride* vs *T. virens*. Left Venn diagram showing up regulated genes by *T. atroviride, T. virens* or both. Middle. Comparison of frequency distribution of up-regulated genes by *T. atroviride* and *T. virens* Right. Results T-test of upregulated genes. The yellow dots represent the genes induced by *T. atroviride* while the pink dots were induced by *T. virens*. (**D**) Comparison of genes down-regulated in *A. thaliana* by *T. atroviride* vs *T. virens*. Left. Venn diagram showing down regulated gens by *T. atroviride, T. virens* or both. Middle. Comparison of frequency distribution of down regulated genes by *T. atroviride* and *T. virens* Right. Results T-test of downregulated genes. The yellow dots represent the genes repressed by *T. atroviride* while the pink dots were repressed by *T. virens*.

To determine the transcriptional response of *Arabidopsis* to *T. atroviride* and *T. virens*, we inoculated the roots of *A. thaliana* seedlings with the fungi and performed RNA-seq analyses using 27 libraries. Quality reports of the sequenced reads showed that all libraries were high quality, exhibiting a Phred > 30 (Fig. S1A). Therefore, we mapped all reads to the reference concatenated genome. On average, more than 95% of the sequencing reads mapped to the reference genome for each library (Fig. S1B). The differential expression analysis showed that 256 and 263 genes were upregulated in *A. thaliana*, whereas 49 and 126 were downregulated by *T. atroviride* and *T. virens* respectively (Figs. 1 and S2; Table S1). Intriguingly, no significant statistical differences (α = 0.05) were found in in the upregulation of genes by *T. atroviride* or *T. virens.* However, *T. virens* was more effective in repressing genes in *A. thaliana* (*p-*val = 0.02, significance 95%) (Fig. 1C,D; Table S1; Fig. S2).

We observed that 138 genes increased their expression in response to both *Trichoderma* species. In addition, 118 and 125 genes were upregulated only in response to *T. atroviride* or *T. virens*, respectively (Figs. 1C,D; S2; Table S1). Using the logFC of the group of 138 upregulated genes in both interactions, applied a *t*-test to determine if any of the two *Trichoderma* species was more effective in modulating differential expression in the plant (Fig. 1C,D; Fig. S2a). The *t*-student test suggested that both fungi similarly modulate differential gene expression (*p-*val = 0.055, significance 95%). Similarly, we observed 37 genes that decreased their expression in response to both *Trichoderma* species. In addition, whereas 12 genes decreased their expression in response to *T. atroviride* and 89 in response to *T. virens* (Fig. 1B,D; Table S1; Fig. S2). The *t*-student test for downregulated genes suggested a difference in gene repression, with *T. virens* being more effective than *T. atroviride* (*p*-val = 0.02, significance 95%).

**Figure 2:**
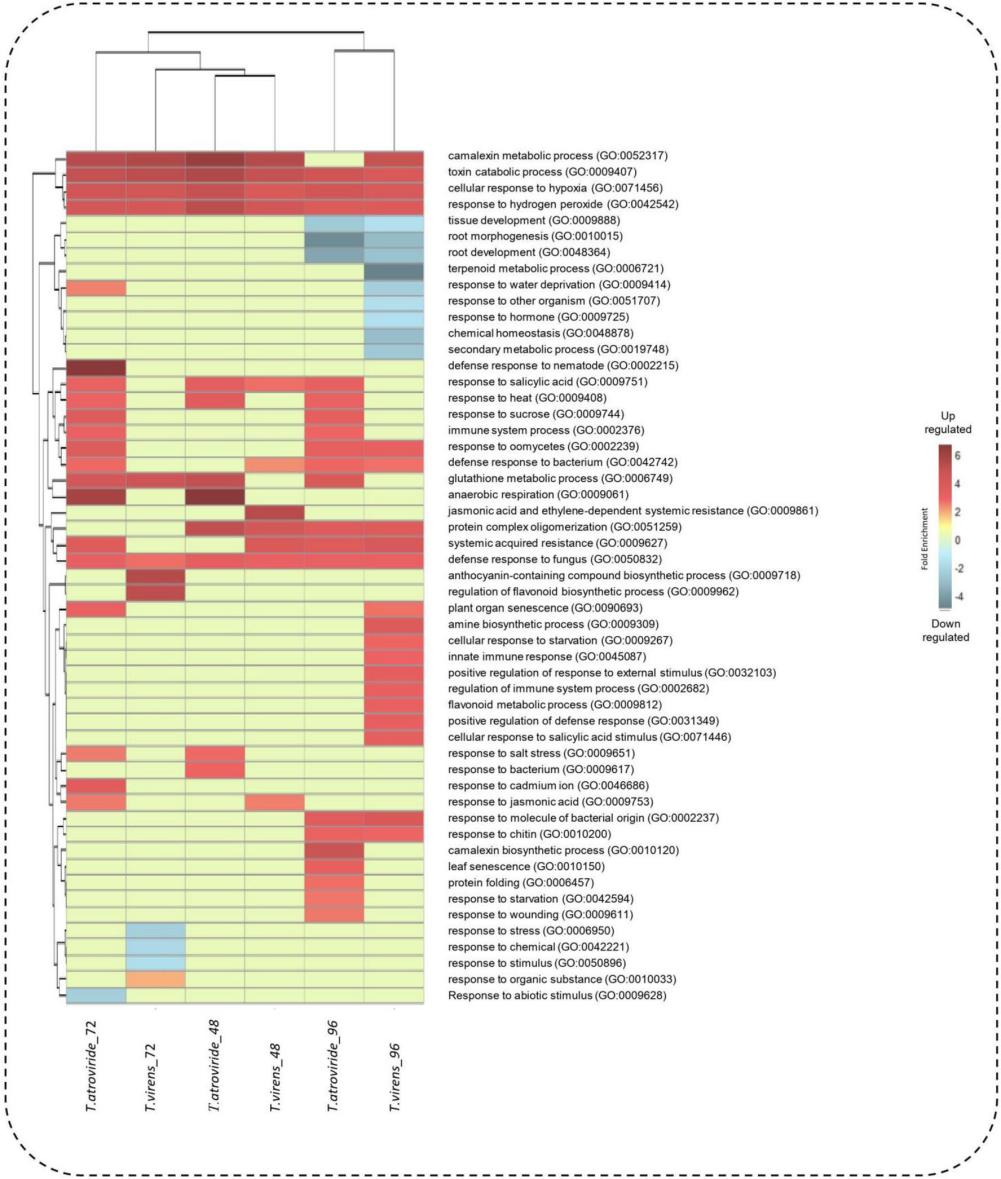
Gene ontology enrichment analysis. Biological process that were differentially regulated in *Arabidopsis thaliana* during its interaction with *Trichoderma* spp. Each column represents a contrast of differential expression, and each row represents the gene ontologies in terms of the biological processes involved. The color gradient goes from blue to red for downregulated and upregulated biological processes, respectively.

The Gene Ontology (GO) enrichment analysis showed the induction of phytohormone pathways such as ethylene, jasmonate, and salicylic acid (Fig. 2; Table S2). Additionally, the induction of mechanisms related to plant defense, such as acquired systemic resistance (GO:0009627), immune system process (GO:0002376), and response to molecule of bacterial origin (GO:0002237), among others, was observed (Fig. 2, Table S2). Mechanisms involved in the regulation of reactive oxygen species scavenging mechanisms, such as the glutathione metabolic process (GO:0006749) and response to hydrogen peroxide (GO:0042542), were also enriched. Finally, among the repressed GO terms: root development (GO:0048364), root morphogenesis (GO:0010015) and chemical homeostasis (GO:0048878) (Fig. 2, Table S2).

### Differential network analysis

**Figure 3:**
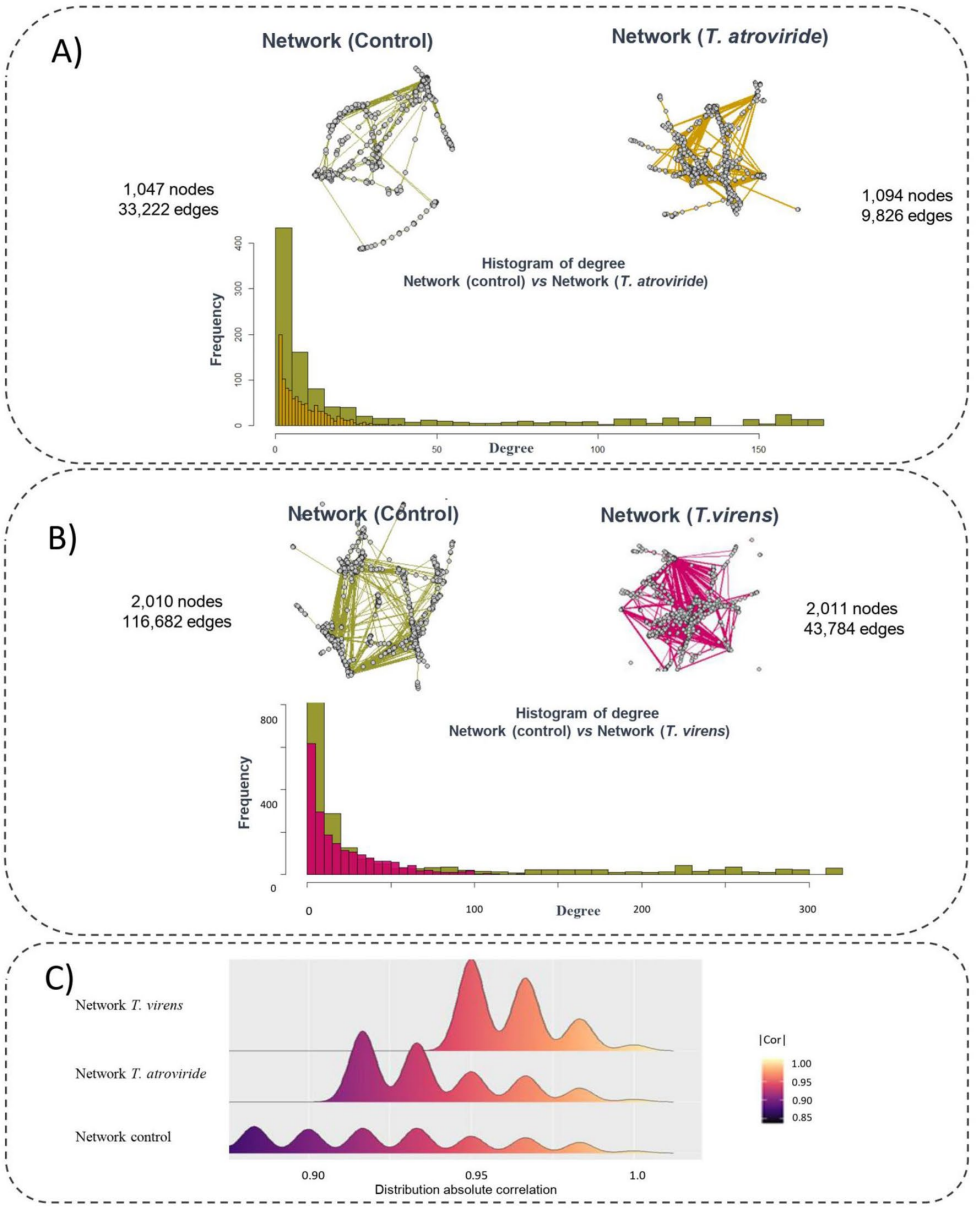
Differential network analysis. (**A**) Co-expression networks under control conditions (green edges), and in interaction with *T.atroviride* (yellow edges) and bottom histogram of degree. (**B**) Co-expression networks under control conditions (green edges), and in interaction with *T. virens* (pink edges) and bottom histogram of degree. (**C**) Correlation distribution analysis for the control, *T. atroviride* and *T. virens* networks. The color gradient goes from black to light yellow for low values and high values of correlation respectively. The graph presents the absolute value of correlation.

As expected, the differential network analysis showed changes in the topology of the interaction networks (network_(*T. atroviride)*_ and network_*(T. virens)*_) against their controls (Fig. 3A,B), probably because of the association of genes under different conditions. When the connectivity of the nodes using the degree as a metric was analyzed, the control networks exhibited a right asymmetry in both cases that extended to 150 and 300 degrees for the *T. atroviride* and the *T. virens* network, respectively. Although the control and the stimulus networks were built with the same nodes, we observed that the control networks had a much greater number of edges in both cases. The *T. atroviride* network contained 9826 edges, whereas its control presented 33,222. On the other hand, the *T. virens* network showed 43,784 edges, while its control had 116,682.

The evaluation of the weights of edges for each network and their respective controls showed that the edges of the interaction networks have greater weight than the edges of the control networks (Fig. 3C). The edges of the *T. virens* network have a correlation coefficient (r) greater than r_s_≥0.95. Similarly, the edges of the *T. atroviride* network have an r_s_≥0.9; finally, the edges of the control networks have an r_s_⩾0.85.

### Gene co-expression network analysis

We generated two gene co-expression networks of *A. thaliana* when stimulated with *T. atroviride* or *T. virens.* The resulting networks consisted of 1094 and 2011 nodes for *Arabidopsis*_*(T. atroviride*)_ and *Arabidopsis*_*(T. virens*)_, respectively (Fig. S4a,b). The networks were simplified, as explained in Fig. S4.

**Figure 4:**
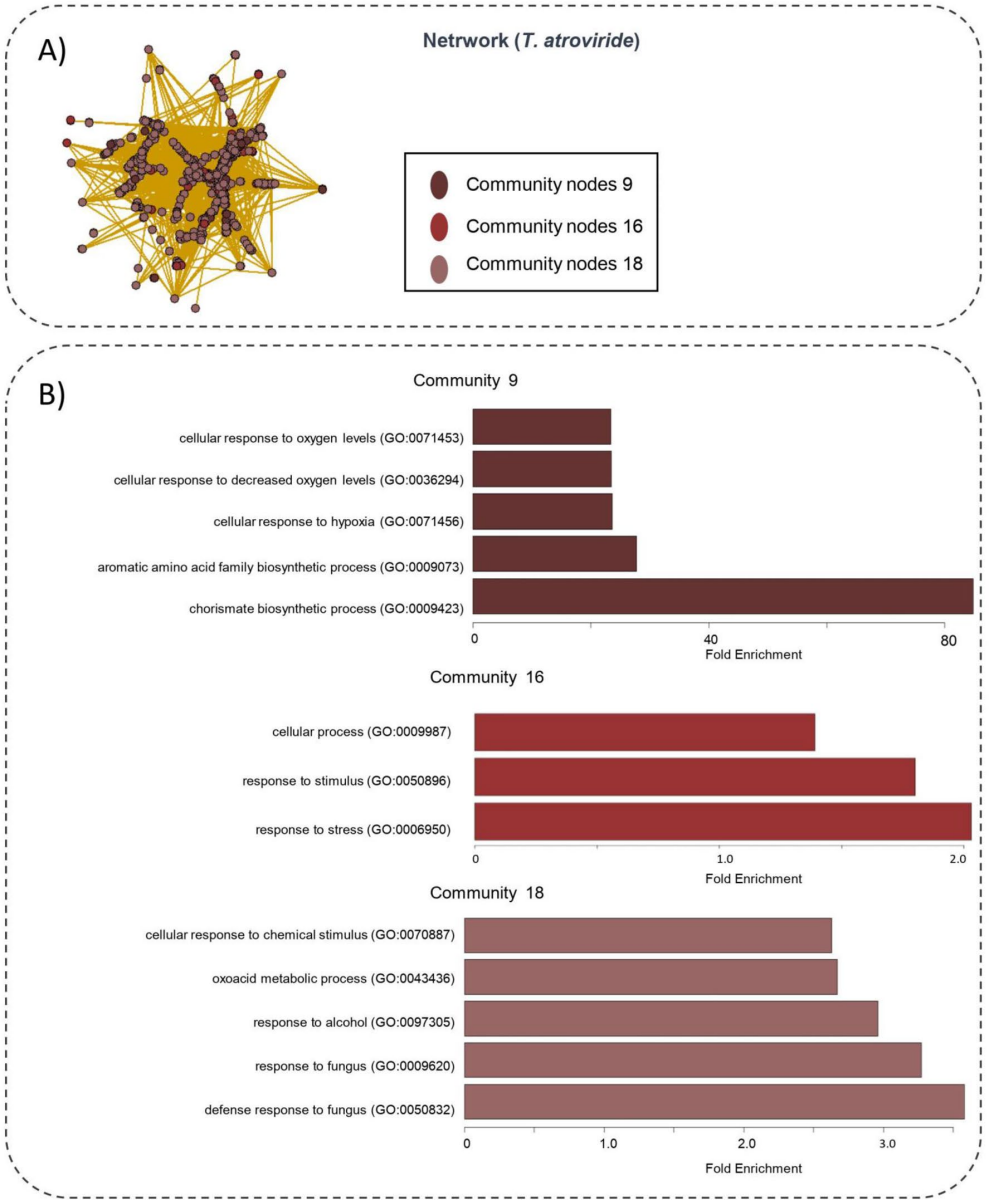
(**A**) Communities of co-expression genes network *A.thaliana*_*(T.atroviride*)_. Communities were identified using the cluster_walktrap(x) algorithm of the igraph package. Each community is represented by a color. (**B**) Functional enrichment analysis. The dark-brown color represents the community 9 (Ca9), the brown color represents the community 16 (Ca16) and the color light-brown represents the community 18 (Ca18).On the y-axis the enriched gene ontologies are shown on the x axis the fold enrichment is observed.

**Figure 5:**
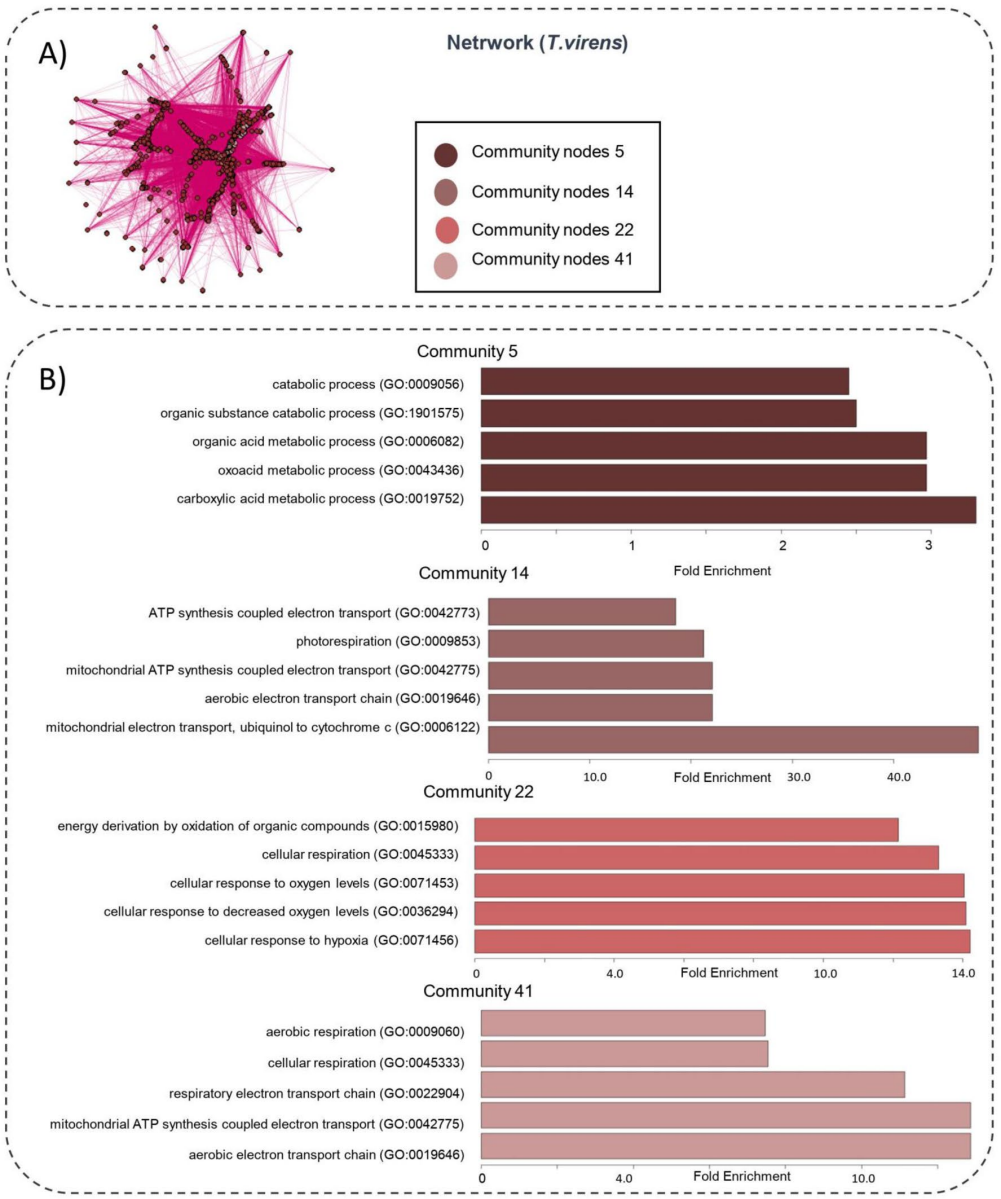
(**A**) Communities of co-expression genes network *A.thaliana*_*(T.virens)*_. Communities were identified using the cluster_walktrap(x) algorithm of the igraph package. (**B**) Functional enrichment analysis. The dark-brown and light-brown bars represent the Cv5 and Cv14 respectively. The dark-pink and light-pink bars represent the Cv22 and Cv41 respectively. On the y-axis the enriched gene ontologies are shown on the x-axis the fold enrichment is observed.

The community identification analysis found 104 communities for the *A. thaliana*_*(T. atroviride)*_ network and 256 for the *A.thaliana*_*(T.virens)*_ network (Fig. S5). We focused only on those communities formed by at least 100 nodes and generated a functional enrichment analysis for communities Ca9, Ca16, and Ca18 for *A.thaliana*_*(T. atroviride)*_ and Cv5, Cv14, Cv22 and Cv41 for *A.thaliana*_*(T. virens)*_ (Figs. 4B & 5B; Table S2).

The processes with the most significant enrichment for each community were in the chorismate biosynthetic process (GO:0009423, Fold enrichment = 84.84) for community Ca9, the stress response (GO:0006950, Fold enrichment = 2.0) for community Ca16, and defense response to fungus (GO: 0050832, Fold enrichment = 3.27) for *A.thaliana*_*(T. atroviride)*_. Besides, in the *A.thaliana*_*(T. virens)*_ network, the processes with the greatest enrichment were carboxylic acid metabolic process (GO:0019752, Fold enrichment = 3.4), mitochondrial electron transport, ubiquinol to cytochrome (GO:0006122, Fold enrichment = 45), cellular response to hypoxia (GO:0071456, Fold enrichment = 14.1) and aerobic electron transport chain (GO:0019646, Fold enrichment = 12) for Cv5, Cv14, Cv22 y Cv41 respectively.

Finally, we selected the HUB nodes using a score based on degree, authority score, and closeness to refine the network (Fig. 4A) and selected the best 50 nodes according to this score from each of the communities (Ca9, Ca16, Ca18 (150 total nodes) and Cv5, Cv14, Cv22 y Cv41(200 total nodes). When we removed the HUB nodes from the network (Fig. S6a), we observed that the network becomes less cohesive and an increase in the geodesic path between nodes. On the other hand, when the nodes considered non-relevant are eliminated, their general cohesion measures increased, indicating that our methodology to identify relevant nodes was adequate.

In the HUB nodes, we identified the AT2G47520 *locus* in both networks (*Arabidopsis*_*(T. atroviride*)_ and *Arabidopsis*_*(T. virens*)_), which encodes a transcription factor necessary to cope with hypoxia in *A. thaliana*^28^ (Fig. S7). This transcription factor has two functional domains (AP2/ERF and Cys2) that suggest a dual role: mitigating hypoxia and coping with pathogen stress. The AT2G47520 *locus* interacted with 22 genes in the *Arabidopsis*_*(T. atroviride*)_ network, while in the *Arabidopsis*_*(T. virens*)_ network, it directly connected with 35 genes. Genes related to hypoxia, jasmonate, and ethylene hormonal pathways, defense mechanisms, and uncharacterized proteins were identified within these groups.

### Experimental validation

We focused on validating our transcriptome and hypotheses using the *A.thaliana-T.atroviride* system. However, considering that both networks have the same statistical parameters, we expected them to behave similarly between the high throughput sequencing and the experimental validation.

**Figure 6:**
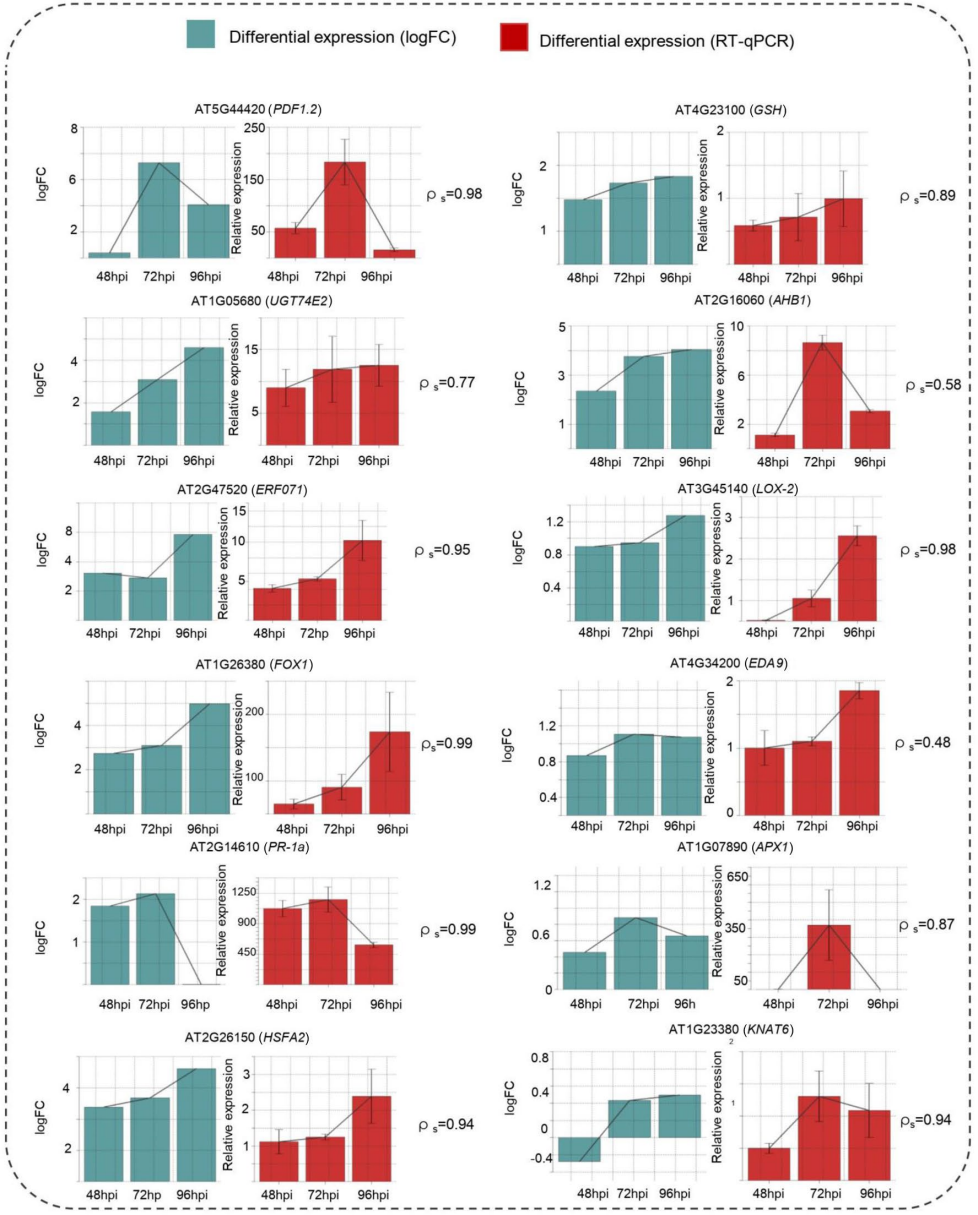
Transcriptome validation. Experimental validation of the transcriptome was performed by RT-qPCR at 48, 72 and 96 hpi. The validation experiment was performed by duplicate. Blue color values obtained of differential expression from the transcriptome; red color values obtained by RT-qPCR. The bars represent the standard deviation (sd) of the average of a triplicate. The correlation coefficient of Sperman (ρ_s_).

To validate our transcriptomic analysis, we selected 12 genes and compared the similarities between our differential expression results obtained by transcriptome and RT-qPCR (Fig. 6). During this analysis, we observed a similar behavior between the expression profiles obtained in the transcriptome and those obtained by RT-qPCR. As expected, in some cases, the same trend was observed for the transcriptomic data and the data obtained by RT-qPCR, as in the cases of the AT5G44420 * (ρ*_*s*_ = *0.98)*, AT2G47520* (ρ*_*s*_ = *0.95)*, AT3G45140 * (ρ*_*s*_ = *0.98) loci*, while in other cases the trend was not exact. Inherently, minimal, and imperceptible changes between the experimental biological replicas explain this phenomenon^29^.

Even though the magnitudes differed since the quantification methods have different principles^30^, we observed similar trends.

## Discussion

### Differential expression analysis

To obtain a general overview of the biological processes involved in *A. thaliana* when in interaction with *Trichoderma* spp., we generated a heat map using differential expression contrasts and GO enrichment analysis for each time point (48, 72, and 96 hpi) (Fig. 2; Table S2). Figure 2 shows the biological processes that were differentially expressed in *A. thaliana* during its interaction with *T. atroviride* or *T. virens*, including mechanisms related to hormonal pathways such as camalexin metabolic process (GO:0052317), response to salicylic acid (GO:0009751), camalexin biosynthetic process (GO:0010120), and response to jasmonic acid (GO:0009753). In addition, defense mechanisms to other organisms including systemic acquired resistance (GO:0009627), response to molecule of bacterial origin (GO:0002237), response to oomycetes (GO:0002239), defense response to fungus (GO:0050832), immune system process (GO:0002376), defense response to nematode (GO:0002215), innate immune response (GO:0045087), and jasmonic acid and ethylene-dependent systemic resistance (GO:0009861). Finally, mechanisms that help alleviate reactive oxygen species were enriched (glutathione metabolic process (GO:0006749), response to hydrogen peroxide (GO:0042542). These results explain well the phenotype observed in the plants after their interaction with *Trichoderma* previously published by different groups^12,31–33^.

It has been reported that different species of *Trichoderma* affect the mechanisms of induced systemic resistance (ISR) in plants, which is related to the jasmonic acid/ethylene signaling pathways^12^. To contrast the induction of ISR by *T. virens* or *T. atroviride*, we visualized (Fig. S3) the differential expression of some relevant genes for this biological process. The *LOX2* and *PDF1.2* genes are ISR marker genes. Despite having no statistical significance (*p*-val = 0.1 & *p*-val = 0.2, respectively), when we performed a Wilcoxon test, we observed that *T. virens* was more efficient for the induction of marker genes (Fig. 3S). Besides, the *WRKY51* (AT5G64810), a JA-regulated gene, showed a lower induction by *T. virens* than that by *T. atroviride* (Fig. S3). The transcription factor WRKY51 was reported by Yan et al.^32^ as a negative regulator of the jasmonate pathway, which forms a protein complex with the JAV1 and JAZ8 proteins. This protein complex (JAV1-JAZ8-WRKY51) controls the biosynthesis of JA so that the plant can defend itself from insect attack^32^. Our results show that *T. virens* is more effective in inducing ISR than *T. atroviride*. Our results agree with those reported by Salas-Marina *et al*^15^, where they observed that *T. virens* had an enhanced effect on the induction of resistance against the necrotrophic fungus *Botrytis cinerea* in tomato plants (*Solanum lycopersicum*).

On the contrary, the ontological term development of the root (GO:0048364) was repressed in both cases after 96 hpi; these data agree with what was reported by Garnica‐Vergara et al.^33^, where they observed an inhibition of primary root growth and induction of lateral root formation in *A. thaliana* after its treatment with *T. atroviride*. Garnica‐Vergara mentions that the plant modifies the architecture of the root due to a blockage in auxin signaling, highlighting the participation of indole-3-acetic acid (IAA). Among the genes found in this group was AT5G56320, which codes for a plant expansin that causes the expansion of cell walls in plants and is known to promote the growth of lateral roots. Additionally, we observed repression of the transcription factor LRL3 (AT5G58010) involved in root hair development. These facts show the complex interaction between plant hormones and their repercussions on the phenotype^33^.

### Differential network analysis

The plant stress response is characterized by an intricate network of signaling cascades that trigger transcriptional reprogramming^34^. To analyze the behavior of genes in *A. thaliana* under control conditions and under stimulus conditions, we performed differential network analysis (Fig. 3A,B). The differential network analysis showed a change in the topology of the interaction networks (network_(*T. atroviride)*_ and network_*(T.virens)*_) against their controls. This fact represents the association behavior of genes under different conditions. Besides, the connections between the nodes were reconfigured, as seen in the histograms in Fig. 3A,B, where the control networks had more edges (33,222 and 116,682 for control-network_(*T. atroviride)*_ and control-network_*(T. virens)*_ respectively) than the interaction networks which had 9826 and 43,784 edges for network_(*T. atroviride)*_ and network_*(T.virens)*_ respectively. This fact suggests that the association between *A. thaliana* genes changes when *Trichoderma* colonizes the plant.

The control network is denser and more connected with edge density = 0.06. In contrast, the *T. atroviride* and *T. virens* networks had edge densities of 0.008 and 0.007, respectively, when we filtered the correlation matrix with a *p*-val = 0.001. Even though the interaction networks have fewer edges than the control network they showed the highest correlation values higher than 0.9 and 0.95 for *T. atroviride* and *T. virens,* respectively. In contrast, the control network has correlation values ​​higher than 0.8 (Fig. 3C). Taken results show that although interaction networks have fewer connections than the control, they are more robust and have higher correlations than their control networks. Thus suggesting a reconfiguration of the genetic transcriptional landscape of the plant when subjected to an external stimulus.

### Gene co-expression network analysis

Here, we generated two gene co-expression networks of *A. thaliana* when stimulated with *T. atroviride* or *T. virens,* and these were simplified as explained below (Fig. 4S). From the transcriptome data, we generated the correlation matrices. The resulting networks consisted of 1094 and 2011 nodes for *Arabidopsis*_*(T. atroviride*)_ and *Arabidopsis*_*(T. virens*)_, respectively (Fig. S4a,b).

An important step in network analysis is to know how the nodes group in communities. Newman and Girvan^35^ defined a community as natural divisions of network nodes into densely connected subgroups. For this purpose, we used the algorithm developed by Pons & Latapi^36^. Our analysis identified 104 communities for the A*. thaliana*_*(T. atroviride)*_ network and 265 for the *A.thaliana*_*(T. virens)*_ network (Fig. S5). However, only communities Ca9, Ca16, and Ca18 for *Arabidopsis*_*(T. atroviride*)_ and Cv5, Cv14, Cv22, and Cv41 for *Arabidopsis*_*(T. virens*)_ were formed with a large number of nodes (Figs. 4, 5).

Due to the nature of the gene co-expression phenomenon, it is important that the genes form a compact and densely interconnected structure^37^. An adequate synergy between the functioning of the genes or their products (proteins) allows the cell to respond and adapt better to stress. Therefore, we focused only on those communities formed by at least 100 nodes (Figs. 4, 5).

Starting from the premise that communities within gene co-expression networks represent biologically functional modules, we generated a functional enrichment analysis for communities Ca9, Ca16, and Ca18 for *A.thaliana*_*(T. atroviride)*_ and Cv5, Cv14, Cv22, and Cv41 for *A.thaliana*_*(T. virens)*_ (Figs. 4B, 5B; Table S2). According to graph theory, the most relevant nodes for a network are those that give the network the most stability^38^. To verify if our methodology to select relevant nodes was adequate, we used the networks formed by 1094 and 2011 nodes (Fig. S6). When we eliminated the relevant nodes, their general cohesion measures decreased with a diameter from 19.14 to 15.85 and 17.29 to 10.85, resulting in a much more compact and stable network. These results showed that our methodology to identify relevant nodes was adequate.

The GO results by community analysis (Figs. 4, 5), such as chorismate biosynthetic process (GO:0009423), defense response to fungus (GO:0050832) or aerobic electron transport chain (GO:0019646), agree with what has been reported. However, studies on the role of hypoxia during the plant-*Trichoderma* interaction are limited. In this sense, plants induce hypoxia-related genes during immersion, flooding, or when growing at high altitudes as the partial pressure of molecular oxygen decreases; however, little is known about the significance of hypoxia triggered by biotic stress. Thus, we focused on this biological process and its possible role during the *Arabidopsis-Trichoderma* interaction.

Despite the scarce studies on hypoxia during plant-microorganism interactions, there is experimental evidence indicating that hypoxia is a common phenomenon during the process. Establishing a symbiotic relationship with nitrogen-fixing rhizobia in legumes requires hypoxic conditions since nitrogenase activity, which can fix N_2_, is inactivated by free O_2_. In other plant species, hypoxia-sensitive genes are induced in stems infected by *Agrobacterium tumefaciens*, suggesting that hypoxic conditions are generated at sites of infection by these microorganisms^39,40^ Although the plant-microorganism interaction largely depends on the lifestyle of the microbe, these studies show that hypoxia exists during the plant colonization event. However, to our knowledge, *Trichoderma* has not been reported to generate hypoxia in its hosts. Based on our results, we hypothesize that *Trichoderma* triggers hypoxia in the host and enhances defense mechanisms against pathogens by stabilizing a family VII transcription factor (ERF-VII) that responds to ethylene.

The association between communities Ca9 and Ca18 makes biological sense since it is known that plants obtain their energy during hypoxia from ATP alcoholic fermentation pathway^41^. On the other hand, the association between communities Cv14 and Cv22 in the *A.thaliana*_*(T. virens)*_ network supports what was reported by^28^, who observed a functional relationship between the mitochondrial electron transport chain and hypoxia after evaluating the functioning of mitochondrial oxidases in different oxygen concentrations. Besides, the network and functional enrichment analyses suggest that hypoxia may play a relevant role in the defense response against fungi (Figs. 4, 5).

To retrieve information, we searched for all the genes with an ontological term related to hypoxia within the general network (1094 and 2011 nodes; Fig. S4). We identified the AT2G47520 *locus* in both networks (*Arabidopsis*_*(T. atroviride*)_ and *Arabidopsis*_*(T. virens*)_), which codes for a transcription factor necessary to cope with hypoxia in *A. thaliana*^28^. Interestingly, this transcription factor has an Ethylene-inducible DNA binding domain (AP2/ERF position 49-106 a.a) (Fig. S7a). In addition, it has a cysteine (Cys2) prone to oxidation in its N-terminal (Fig. S7a,c). Therefore, this transcription factor is more stable during hypoxia than under normoxic conditions. It is known that the AP2/ERF domain can interact with the GCC-box promoter regions upstream of genes encoding pathogenesis-related proteins^42^. Therefore, the presence of these two domains (AP2/ERF and Cys2) in AT2G47520 suggests a dual role, first being more stable in hypoxic conditions to activate defense genes further. We analyzed positional weight matrices (PWM) and identified that the transcription factor can bind to the GCC-box (Fig. S7b,d). Brown et al.^43^, observed that deletion or point mutations in the core sequence of the GCC box substantially reduced the plant's ability to respond to JA. On the other hand, Epple & Bohlmann^44^ observed that plants that overexpress the *Thi2.1* gene, a gene with the GCC-box sequence in its promoter, improve resistance against the phytopathogen *Fusarium oxysporum*.

The AT2G47520 *locus* interacted with 22 genes in the *Arabidopsis*_*(T.atroviride*)_ network. AT3G03270, AT4G24110, and AT3G27220 respond to hypoxia. AT1G43800 encodes a protease that participates in ISR, among others. In the *Arabidopsis*_*(T.virens*)_ network, it was directly connected with 35 genes. Within this set of genes is AT2G26020, which encodes the plant defensin *PDF1.2b*, which is involved in defense mechanisms against fungi; AT5G07580, which codes for an ethylene response transcription factor; and AT1G66350, a protein with a role in jasmonic acid signaling. This fact suggests a functional relationship between hypoxia and the plant's defense mechanisms, as well as the participation of hormones in this genetic association.

The idea that plants induce their hypoxic genes and defense mechanisms against pathogens is consistent if we consider that plants suffer constant biotic stress after floods. On the one hand, there is dispersion of pathogens by rain, in addition to the fact that plants exhibit morphological changes after floods that facilitate infection by pathogens, such as change in intracellular pH, change in stomata behavior, and a decrease in hydraulic conductance^45^. These observations suggest that despite being a stressful stage for the plant, hypoxia promotes resistance against certain pathogens. For these reasons, we hypothesize that hypoxia and plant defense mechanisms are interconnected through signaling pathways that have not yet been explored and could involve signaling pathways related to the synthesis of JA. Yuan et al.^46^ reported that after hypoxia, there is a rapid accumulation of (JA) and an induction of genes involved in the synthesis of JA. In addition, applying exogenous methyl jasmonate (MeJA) improved tolerance to posthypoxic stress in *A. thaliana* plants. A recent work^47^ mentions that these two stresses occur sequentially or simultaneously in plants. During evolution, plants developed efficient strategies to respond to stimuli with the minimum energy expenditure through multiple functions of a single gene.

### Experimental validation

To validate our transcriptomic analysis, we selected 12 genes, and we collected samples at times 48, 72, and 96 hpi. In this sense,10 genes were chosen from the main network, selecting those that will help us to solve future investigations and hypotheses and that represent different communities of the general network (1094 nodes). Additionally, 2 extra genes *PR-1a* (AT2G14610) and *LOX2* (AT3G45140) were selected, which were not in the network but are commonly used as marker genes to validate the induction of systemic responses against pathogens. We compared the similarities between the differential expression results obtained by transcriptome and RT-qPCR (Fig. 6). During this analysis we observed a similar trend between the expression profiles obtained in the transcriptome, RNA-seq, and those obtained by RT-qPCR. In some cases, the exact same trend is observed for the transcriptomic data and the data obtained by RT-qPCR, as in the cases of the AT5G44420 *(ρ*_*s*_ = *0.98)*, AT2G47520 *(ρ*_*s*_ = *0.95)*, AT3G45140 *(ρ*_*s*_ = *0.98) loci*, while in other cases the trend is not exact (for example, AT2G16060 *ρ*_s_ = 0.58, AT4G34200 with *ρ*_s_ = 0.48 and AT1G05680 * ρ*_s_ = 0.77). This fact is completely natural and expected, inherently there are minimal and imperceptible changes between the experimental biological replicas that explain this phenomenon^29^.

## Conclusions

Our current study shows gene co-expression network analysis as a methodology that enables us to observe how genes associate into biologically functional modules in *Arabidopsis* that respond to an interaction with mutualistic fungi.

During plant-fungus interaction, there is a functional interconnection between hypoxic mechanisms and the plant defense mechanisms. However, we have carefully mentioned that we have only generated hypotheses requiring further experimental evidence. In the future, we will experimentally study the possible interconnection between hypoxia and defense mechanisms during the plant-fungus interaction. These interconnections will be identified in mutant plants in selected genes of our analysis. These experiments will allow us to clarify this complex mechanism that has led plants to respond appropriately to different types of stress by expending the minimum energy and anticipating future challenges that could end in plant death.

The AT2G47520 *locus* encodes a transcription factor with protein domains (AP2/ERF position 49-106 a.a. and Cys2) with dual function to counteract hypoxia and pathogens.

## Materials and methods

### Plant and fungal growth conditions

Columbia ecotype (Col-0) of *Arabidopsis thaliana* plants, *T. atroviride* (IMI206040), and *T. virens (*Gv29-8)^48^ fungus as colonizing were used in this study. *Trichoderma* spp. was grown in potato dextrose agar (PDA) (Becton, Dickinson & Co) and incubated at 28 °C under 12/12 light/dark cycles. Seeds of *A. thaliana* Col-0 were sterilized by two consecutive washings using a 75% ethanol solution for 3 min under constant shaking at a speed of 1800 rpm using a MixMate (Eppendorf SE, 5353, Germany) and with 20% chlorine solution for 7 min. Subsequently, the seeds were rinsed with sterile distilled water. *A. thaliana* seeds were germinated in Murashige and Skoog medium [1x] containing 10% sucrose (PhytoTechnology Laboratories, USA). Subsequently, the seeds were incubated for ≈12 days at 22 ± 1 °C with a regime of 16 h of light and 8 h of darkness^49^.

## *Arabidopsis thaliana*–*Trichoderma* spp. Interaction assay

The 12-day-old *Arabidopsis* plants were inoculated with *T. atroviride or T. virens* mycelium plugs (d = 5 mm). Uninoculated plants were used as controls. Subsequently, biological leaf and root samples were collected at 48, 72, and 96 h after inoculation (hpi) for both the control groups and the different interactions. The collected samples were frozen in liquid nitrogen until their use. A new *Arabidopsis-Trichoderma* interaction was carried out to validate the transcriptome under the same conditions.

### Total RNA extraction, library preparation, and sequencing

Total RNA was extracted using the method reported by Ferdous et al.^50^ with slight modifications. Briefly, frozen samples were macerated in extraction buffer. The cell lysate was carried out using the cationic detergent hexadecyltrimethylammonium bromide (CTAB). The recovery of the organic phase was carried out with 150 μL of phenol pH 8, and chloroform-isoamyl alcohol (24:1). Finally, the RNA was precipitated with isopropanol at − 20 °C overnight. The samples were treated with DNAse to remove contaminating DNA, using the commercial kit TURBO DNA-free™ KIT (Ambion, Life Technologies). RNA was quantified in an Epoch Microplate Spectrophotometer (BioTek®) at an OD of 260 and 280 nm (nanometers). RNA integrity was visualized by denaturing agarose gel electrophoresis [0.8%], and the samples were inspected using Agilent 2100 Bioanalyzer technology (Agilent Technologies, Inc., USA). Finally, 27 libraries were generated and sequenced in a paired manner with the Illumina Hiseq 2500 technology (Illumina Inc., Hiseq 2500, USA) by the National Laboratory of Genomics for Biodiversity, LANGEBIO, Irapuato Mexico.

### Data collection and processing

A transcriptome analysis of the 27 libraries of *A. thaliana* interaction with *T. atroviride* or *T. virens* at 48, 72, and 94hpi and their respective controls without the fungi was performed. Each interaction (*Arabidopsis*_*(T. atroviride*)_ and *Arabidopsis*_*(T. virens*)_) and controls included three independent biological replicates. Gene expression datasets were processed using the R software version 4.2.1 (2022–06-23 ucrt) (https://www.R-project.org/) and Rstudio Team (2022). Rstudio: Integrated Development for R. Rstudio, PBC, Boston, MA URL (http://www.rstudio.com/) version 2022.7.1.554.

### Quality analysis and mapping of the reads to the reference genome

Langebio provided a quality analysis of the FASTQ files. All reads presented a Phred quality score higher than 30, which was considered as high quality and used for mapping. Reads were mapped to a concatenated reference genome (*A. thaliana-T. atroviride-T. virens*). The concatenated genome was constructed taking the reference genomes reported by TAIR (The Arabidopsis Information Resource) version TAIR10 and for *T. atroviride* and *T. virens* reported by Ensembl Fungi (https://fungi.ensembl.org) version IMI_206040_v2.0 and ASM17099_v1 respectively. Mapping of the fragments was carried out using the HISAT2 software with default parameters for paired-end reads and a bash script^51^. Mapped fragments were quantified with the featureCounts tool from the SubRead package^52^. With these quantifications, a counts matrix was generated with an in-house R script.

### Differential expression analysis

The differential gene expression analysis was performed with the edgeR package^53^ using the count matrix. Normalization factors and variability were calculated with the *calcNormFactors(x)* and *estimateDisp(x)* functions, respectively. Subsequently, the data were fitted with the function *glmFit(x)*. Contrasts were performed with a likelihood ratio test, *glmQLFTest(x)* function, to identify changes in gene expression under control and plant-microorganism interaction conditions for each time (48, 72, and 96 hpi), with which a differential expression matrix was generated for each contrast. We keep differential expressed genes with a cutoff of FDR < 0.001 and a logFC > 1.5 or logFC < -1.5.

### Differential network analysis

Two gene co-expression networks were generated (*Arabidopsis*_*(T. virens*)_ and *Arabidopsis*_*(T. atroviride*)_). All differential expression contrasts (*Arabidopsis*_*(T.virens*)_ and *Arabidopsis*_*(T.atroviride*)_) results were used to keep differentially expressed genes, using a false discovery rate (FDR) of FDR = 0.001. The control networks were built using the same genes that were present in the interaction networks (*Arabidopsis*_*(T.atroviride*)_) 1094 and *Arabidopsis*_*(T.virens*)_, 2011 nodes) and the control libraries were used (*A. thaliana* without interaction). The control networks were filtered with an FDR = 0.001. A correlation test was performed with the cor.test(x) functions from the R base package. The correlations were filtered using a threshold of FDR = 0.001, and the correlation significance threshold was *ρ*_s_ = 0.8.

## Construction and analysis of co-expression networks

Two gene co-expression networks were generated (*Arabidopsis*_*(T. virens*)_ and *Arabidopsis*_*(T. atroviride*)_). Each of the differential expression contrasts (*Arabidopsis*_*(T. virens*)_ and *Arabidopsis*_*(T. atroviride*)_) results were used to keep differentially expressed genes, using a false discovery rate (FDR) of FDR = 0.001. The resulting genes were used to generate the correlation matrix using Spearman's method defined in the *cor(x)* function from the R base package. A correlation test was performed for each correlation with the cor.test(x) functions from the R base package. For each correlation test, we obtained a Spearman correlation coefficient ${\rho }_{s}$ and a *p-*value. For all the correlation tests we obtained the FDR by using the p.adjust(x) frunction from R stats package. The correlations were filtered using a threshold of FDR = 0.001. The resulting correlations were used to build gene co-expression networks. The analysis and visualization of the network were carried out using the igraph package with the R programming language^54^^.^ The correlations were filtered using a threshold of FDR = 0.001, and the correlation significance threshold was *ρ*_s_ = 0.8.

The two co-expression networks *Arabidopsis*_*(T. virens)*_ and *Arabidopsis*_*(T. atroviride)*_ formed by 1094 and 2011 nodes, respectively. Communities were identified for each of the networks (*Arabidopsis*_*(T. virens*)_ and *Arabidopsis*_*(T. atroviride*)_) using the algorithm developed by Pons and Latapy^36^ with the function *cluster_walktrap(x)* from the igraph package*,* with default parameters. Subsequently, the communities with more than 100 nodes were compared with each other based on the degree, authority score, and closeness metrics. Finally, an analysis of functional enrichment was carried out in the identified communities using the Protein analysis through evolutionary relationships (PANTHER, https://www.panther.db.org) server version 17.0 (released 2022-02-22)^55^ which implements an additional statistical test method (Fisher's exact test) for the PANTHER overrepresentation test, as well as the Benjamini–Hochberg False Discovery Rate for multiple testing correction.

For selecting HUB nodes, we use a score defined as the product of centrality, closeness, and authority measures. Using the score in decreasing order, we used the best 50 nodes for each community and generated two sub-networks *Arabidopsis*_*(T. virens)*_ and *Arabidopsis*_*(T. atroviride)*_*.*

A new interaction assay was carried out to validate the transcriptome. Two biological replicates were carried out with three technical replicates in each. For the experimental validation, ten genes were chosen from the main network, selecting those that will help us solve future investigations and hypotheses and represent different communities of the general network (1094 nodes). Additionally, two extra genes PR1a (AT2G14610) and Lox2 (AT3G45140), were selected, which were not in the network but are commonly used as marker genes to validate the induction of systemic responses against pathogens.

Finally, we calculated the Spearman correlation coefficient between the transcriptomic data (logFC) and the data obtained by RT-qPCR.

### Plants guidelines

The authors confirm that the use of plants in the present study complies with international, national, and institutional guidelines.

### Supplementary Information

Supplementary Figures.

Supplementary Tables.

## Data Availability

The raw RNA-seq data can be found in the Gene Expression Omnibus (GEO) database under the series record GSE235893.

## References

[1] Gull A (2019). Biotic and abiotic stresses in plants. Abiotic Biotic Stress Plants.

[2] Hartman S (2019). Ethylene-mediated nitric oxide depletion pre-adapts plants to hypoxia stress. Nat. Commun..

[3] Hermosa R, Viterbo A, Chet I, Monte E (2012). Plant-beneficial effects of *Trichoderma* and of its genes. Microbiology (Reading).

[4] Jones JDG, Dangl JL (2006). The plant immune system. Nature.

[5] Chisholm ST, Coaker G, Day B, Staskawicz BJ (2006). Host-microbe interactions: shaping the evolution of the plant immune response. Cell..

[6] Thomas JD, Thomas RE, Thomas JL (2016). Intracellular innate immune surveillance devices in plants and animals. Science..

[7] Peng Y, van Wersch R, Zhang Y (2018). Convergent and divergent signaling in PAMP-triggered immunity and effector-triggered immunity. Mol. Plant Microbe Interact..

[8] Zhang Y (2010). Control of salicylic acid synthesis and systemic acquired resistance by two members of a plant-specific family of transcription factors. Proc. Natl. Acad. Sci. U.S.A..

[9] Hammond-Kosack KE, Jones JD (1997). Plant disease resistance genes. Annu. Rev. Plant Biol..

[10] Woo SL, Hermosa R, Lorito M, Monte E (2022). *Trichoderma*: A multipurpose, plant-beneficial microorganism for eco-sustainable agriculture. Nat. Rev. Microbiol..

[11] Yang DL (2012). Plant hormone jasmonate prioritizes defense over growth by interfering with gibberellin signaling cascade. Proc. Natl. Acad. Sci. U.S.A..

[12] Salas-Marina MA (2011). Colonization of *Arabidopsis* roots by *Trichoderma atroviride* promotes growth and enhances systemic disease resistance through jasmonic acid/ethylene and salicylic acid pathways. Eur. J. Plant. Pathol..

[13] Schmoll M, Schuster A (2010). Biology and biotechnology of *Trichoderma*. Appl. Microbiol. Biotechnol..

[14] Brooks DM, Bender CL, Kunkel BN (2005). The Pseudomonas syringae phytotoxin coronatine promotes virulence by overcoming salicylic acid-dependent defences in *Arabidopsis thaliana*. Mol. Plant. Pathol..

[15] Salas-Marina MA (2015). The Epl1 and Sm1 proteins from *Trichoderma atroviride* and *Trichoderma virens* differentially modulate systemic disease resistance against different life style pathogens in Solanum lycopersicum. Front. Plant Sci..

[16] Mona SA (2017). Increased resistance of drought by *Trichoderma harzianum* fungal treatment correlates with increased secondary metabolites and proline content. J. Integr. Agric..

[17] Zhang C (2022). A novel salt-tolerant strain *Trichoderma atroviride* HN082102.1 isolated from marine habitat alleviates salt stress and diminishes cucumber root rot caused by Fusarium oxysporum. BMC Microbiol..

[18] Fukushima S, Mori M, Sugano S, Takatsuji H (2016). Transcription factor WRKY62 plays a role in pathogen defense and hypoxia-responsive gene expression in rice. Plant Cell Physiol..

[19] Loreti E, Perata P (2020). The many facets of hypoxia in plants. Plants.

[20] Hattori Y (2009). The ethylene response factors SNORKEL1 and SNORKEL2 allow rice to adapt to deep water. Nature.

[21] Coppola M (2019). Transcriptome and metabolome reprogramming in tomato plants by *trichoderma* harzianum straint22 primes and enhances defense responses against aphids. Front. Physiol..

[22] De Palma M (2019). Transcriptome reprogramming, epigenetic modifications and alternative splicing orchestrate the tomato root response to the beneficial fungus *Trichoderma* harzianum. Hortic. Res..

[23] Serin EAR, Nijveen H, Hilhorst HWM, Ligterink W (2016). Learning from co-expression networks: Possibilities and challenges. Front. Plant Sci..

[24] Pellegrini M, Haynor D, Johnson JM (2004). Protein interaction networks. Expert Rev. Proteomics.

[25] Jiang D (2019). Microbiome multi-omics network analysis: Statistical considerations, limitations, and opportunities. Front. Genet..

[26] Amitai G (2004). Network analysis of protein structures identifies functional residues. J. Mol. Biol..

[27] Cohen, R. & Havlin, S. Structure and robustness of complex networks. In *Complex Networks: Structure, Robustness and Function* 65–95 (2010).

[28] Licausi F (2010). HRE1 and HRE2, two hypoxia-inducible ethylene response factors, affect anaerobic responses in *Arabidopsis thaliana*. Plant J..

[29] Ohmiya H (2014). RECLU: A pipeline to discover reproducible transcriptional start sites and their alternative regulation using capped analysis of gene expression (CAGE). BMC Genomics.

[30] Wang Z, Gerstein M, Snyder M (2009). RNA-Seq: A revolutionary tool for transcriptomics. Nat. Rev. Genet..

[31] Jogaiah S, Abdelrahman M, Tran LSP, Ito SI (2018). Different mechanisms of *Trichoderma virens*-mediated resistance in tomato against Fusarium wilt involve the jasmonic and salicylic acid pathways. Mol. Plant Pathol..

[32] Yan C (2018). Injury Activates Ca^2+^/calmodulin-dependent phosphorylation of JAV1-JAZ8-WRKY51 complex for Jasmonate biosynthesis. Mol. Cell.

[33] Garnica-Vergara A (2016). The volatile 6-pentyl-2H-pyran-2-one from *Trichoderma atroviride* regulates *Arabidopsis thaliana* root morphogenesis via auxin signaling and ETHYLENE INSENSITIVE 2 functioning. New Phytol..

[34] Halder K, Chaudhuri A, Abdin MZ, Majee M, Datta A (2022). Chromatin-based transcriptional reprogramming in plants under abiotic stresses. Plants (Basel).

[35] Newman MEJ, Girvan M (2003). Finding and evaluating community structure in networks. Phys. Rev. E Stat. Nonlinear Soft Matter. Phys..

[36] Pons, P. & Latapy, M. Computing communities in large networks using random walks. In *Lecture Notes in Computer Science (including subseries Lecture Notes in Artificial Intelligence and Lecture Notes in Bioinformatics)* **3733 LNCS**, 284–293 (2005).

[37] Novershtern N (2011). Densely interconnected transcriptional circuits control cell states in human hematopoiesis. Cell.

[38] Ghoshal G, Barabási AL (2011). Ranking stability and super-stable nodes in complex networks. Nat. Commun..

[39] Pucciariello C, Boscari A, Tagliani A, Brouquisse R, Perata P (2019). Exploring legume-rhizobia symbiotic models for waterlogging tolerance. Front. Plant Sci..

[40] Kerpen L, Niccolini L, Licausi F, van Dongen JT, Weits DA (2019). Hypoxic conditions in crown galls induce plant anaerobic responses that support tumor proliferation. Front. Plant Sci..

[41] António C (2016). Regulation of primary metabolism in response to low oxygen availability as revealed by carbon and nitrogen isotope redistribution. Plant Physiol..

[42] Ohme-Takagi M, Shinshi H (1995). Ethylene-inducible DNA binding proteins that interact with an ethylene-responsive element. Plant Cell.

[43] Brown RL, Kazan K, McGrath KC, Maclean DJ, Manners JM (2003). A role for the GCC-box in Jasmonate-mediated activation of the PDF1.2 gene of *Arabidopsis*. Plant Physiol..

[44] Epple P, Apel K, Bohlmann H (1997). Overexpression of an endogenous thionin enhances resistance of *Arabidopsis* against Fusarium oxysporum. Plant Cell.

[45] Choat B, Gambetta GA, Wada H, Shackel KA, Matthews MA (2009). The effects of Pierce’s disease on leaf and petiole hydraulic conductance in *Vitis vinifera* cv. Chardonnay. Physiol. Plant.

[46] Yuan LB (2017). Jasmonate regulates plant responses to postsubmergence reoxygenation through transcriptional activation of antioxidant synthesis. Plant Physiol..

[47] Tang H, Liu H (2021). Roles of single gene in plant hypoxia and pathogen responses. Plant Signal Behav..

[48] Baek JM, Kenerley CM (1998). The arg2 gene of Trichoderma virens: cloning and development of a homologous transformation system. Fungal Genet. Biol..

[49] Estrada-Rivera M (2020). IPA-1 a putative chromatin remodeler/helicase-related protein of *Trichoderma virens* plays important roles in antibiosis against Rhizoctonia Solani and induction of *Arabidopsis* systemic disease resistance. Mol. Plant Microbe Interact..

[50] Ferdous J (2014). A quick DNA extraction protocol: Without liquid nitrogen in ambient temperature. Afr. J. Biotechnol..

[51] Kim D, Paggi JM, Park C, Bennett C, Salzberg SL (2019). Graph-based genome alignment and genotyping with HISAT2 and HISAT-genotype. Nat. Biotechnol..

[52] Liao Y, Wang J, Jaehnig EJ, Shi Z, Zhang B (2019). WebGestalt 2019: Gene set analysis toolkit with revamped UIs and APIs. Nucleic Acids Res..

[53] Robinson MD, McCarthy DJ, Smyth GK (2010). edgeR: A Bioconductor package for differential expression analysis of digital gene expression data. Bioinformatics.

[54] Csárdi, G. & Nepusz, T. The igraph software package for complex network research. (2006).

[55] Thomas PD (2022). PANTHER: Making genome-scale phylogenetics accessible to all. Protein Sci..

