# Supplementary material for: Inferring co-expression networks of Arabidopsis thaliana genes during their interaction with Trichoderma spp
Source: Sci Rep. 2024 Jan 30;14:2466. doi: 10.1038/s41598-023-48332-w (PMC10827721; doi:10.1038/s41598-023-48332-w)
Supplement: Supplementary file 1 — Supplementary Figures. [file 41598_2023_48332_MOESM1_ESM.docx]

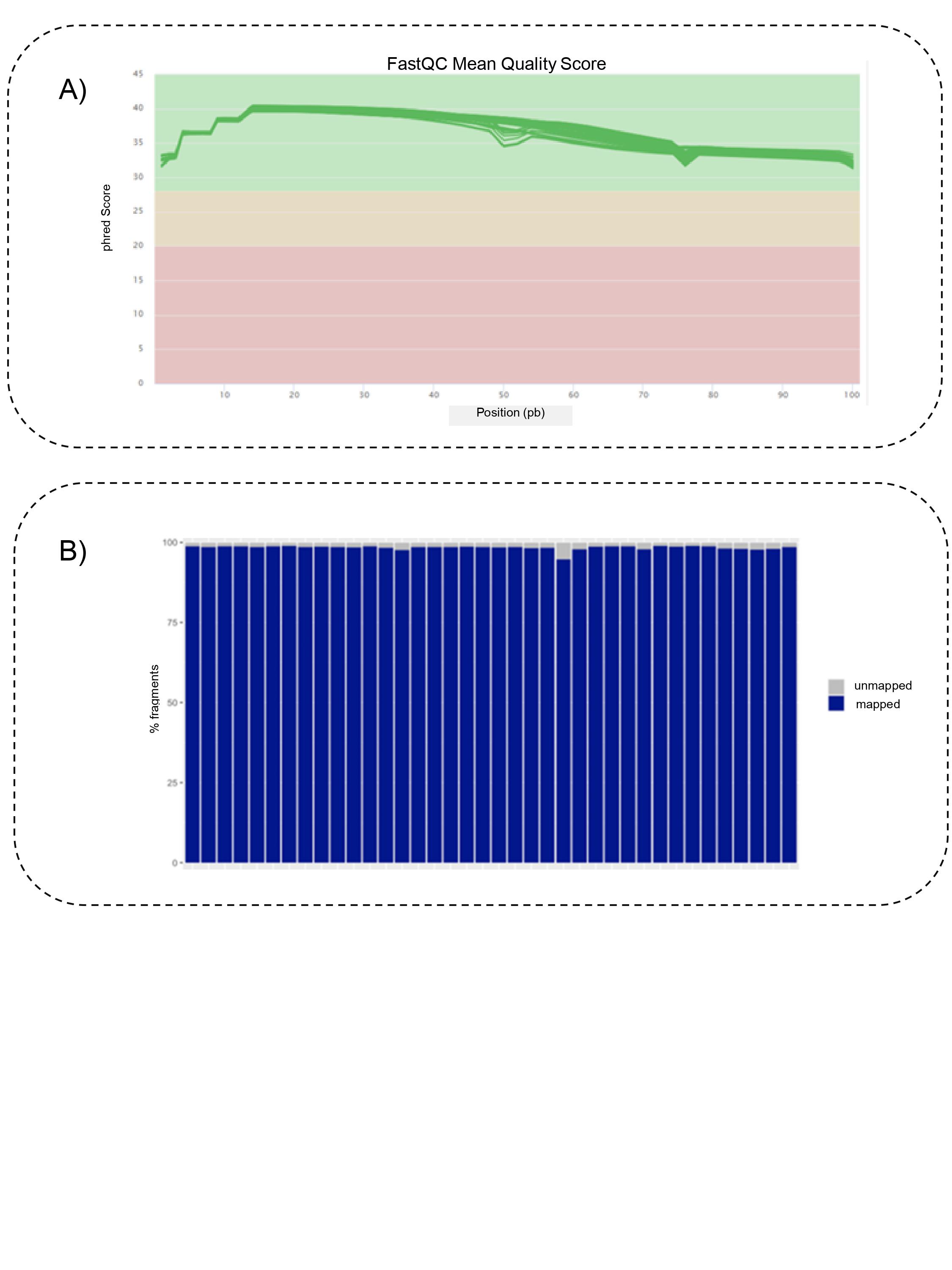


Figure S1. A) Phred quality score graphic. High consistency of a sequenced base is indicated by greater values of Phred. A Phred Score<20 indicates low quality, 25>Phred>20 indicates medium quality and a Phred>35 indicates high quality B) Percentage of reads mapped to the reference genome. Each bar represents a library. The blue color within the bar represents reads mapped to the reference concatenated genome, the gray color within the bar represents the percentage of unmapped reads.


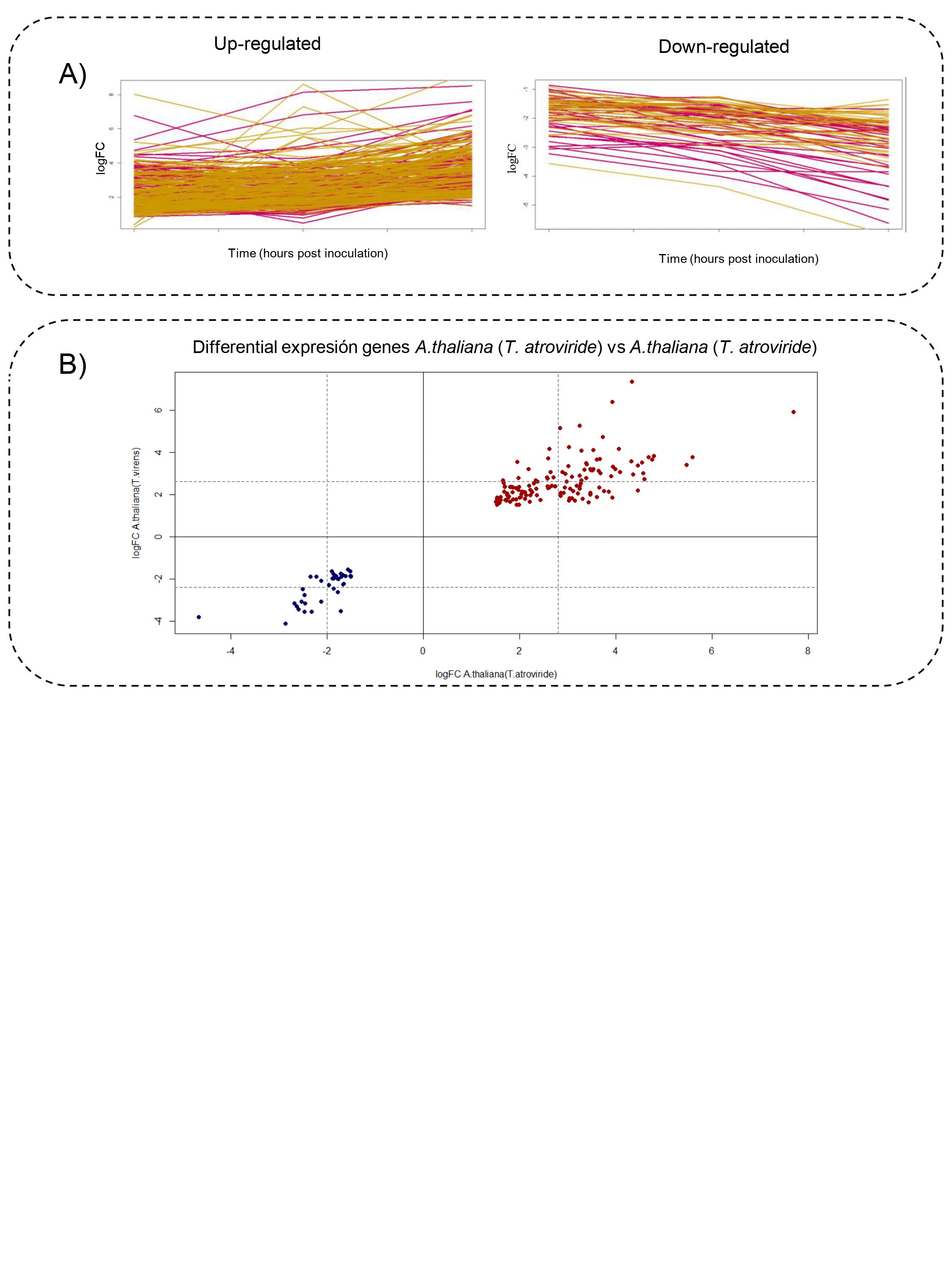


Figure S2. Differential expression analysis. A). Temporal kinetics. On y-axis is show the logFC versus x-axis represents the time after induction (48,72 and 96 hpi), the pink lines represent induction by *T.virens* and yellow lines represent induction by *T. atroviride.* B) Comparison of genes up-regulated and down-regulated in *A. thaliana* by *T. atroviride* vs *T. virens*. On y-axis is show logFC by *T. virens*, x-axis represents logFC by *T. atroviride*. Blue dots represent down-regulated genes and red dots represent up-regulated genes.


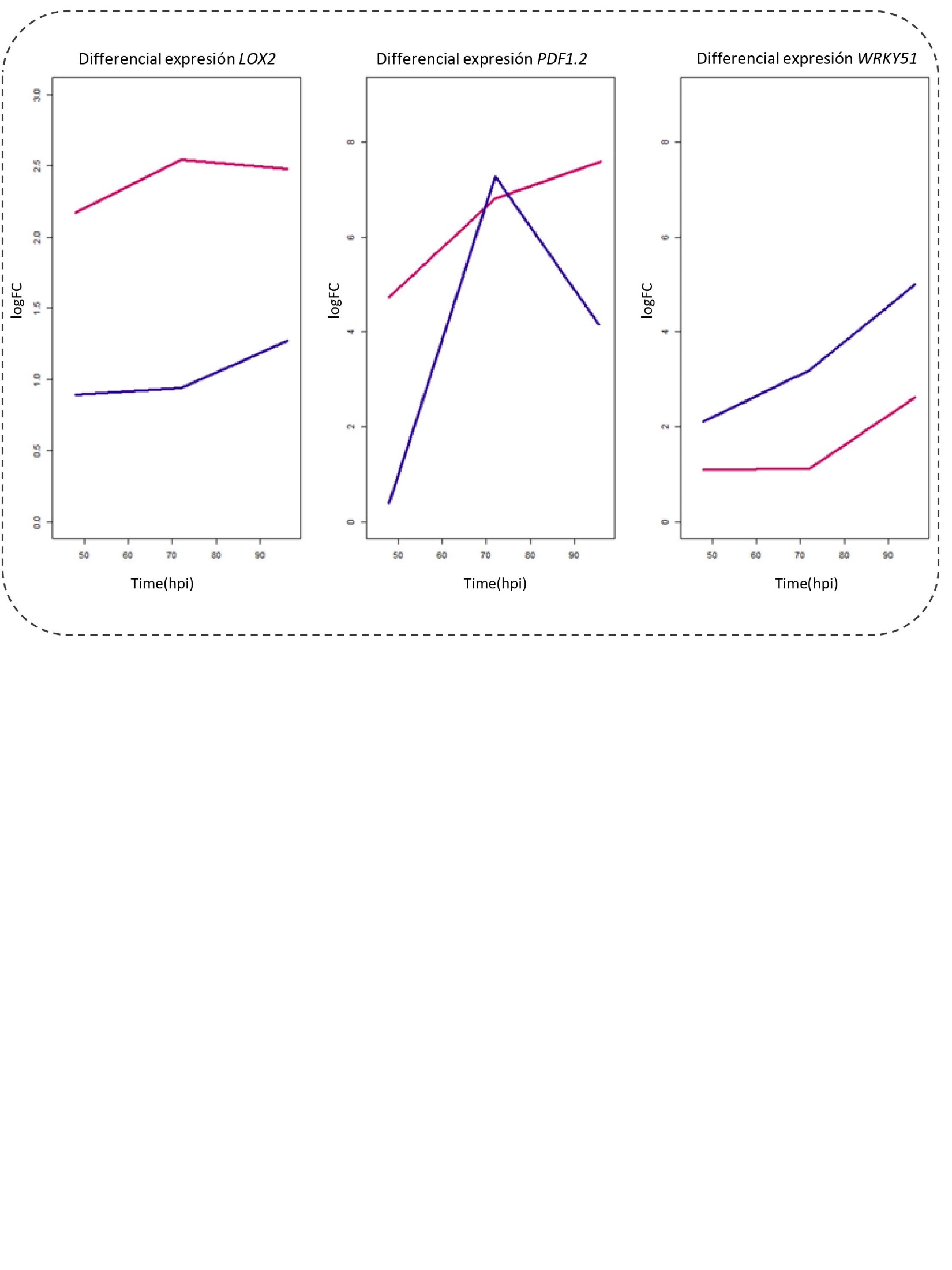


Figure S3. Induction of ISR related genes in *A. thaliana* by *T. atroviride* or *T. virens*. Kinetics of induction of the *LOX2*, *PDF1.2* and *WRKY51* genes. The pink lines represent the genes induced by *T. virens* and the blue lines represent the genes induced by *T. atroviride*. The logFC value was calculated from the contrasts of differential expression using the normalized matrix of counts. Samples were collected at 48, 72 and 96 hours post inoculation with *Trichoderma* spp.


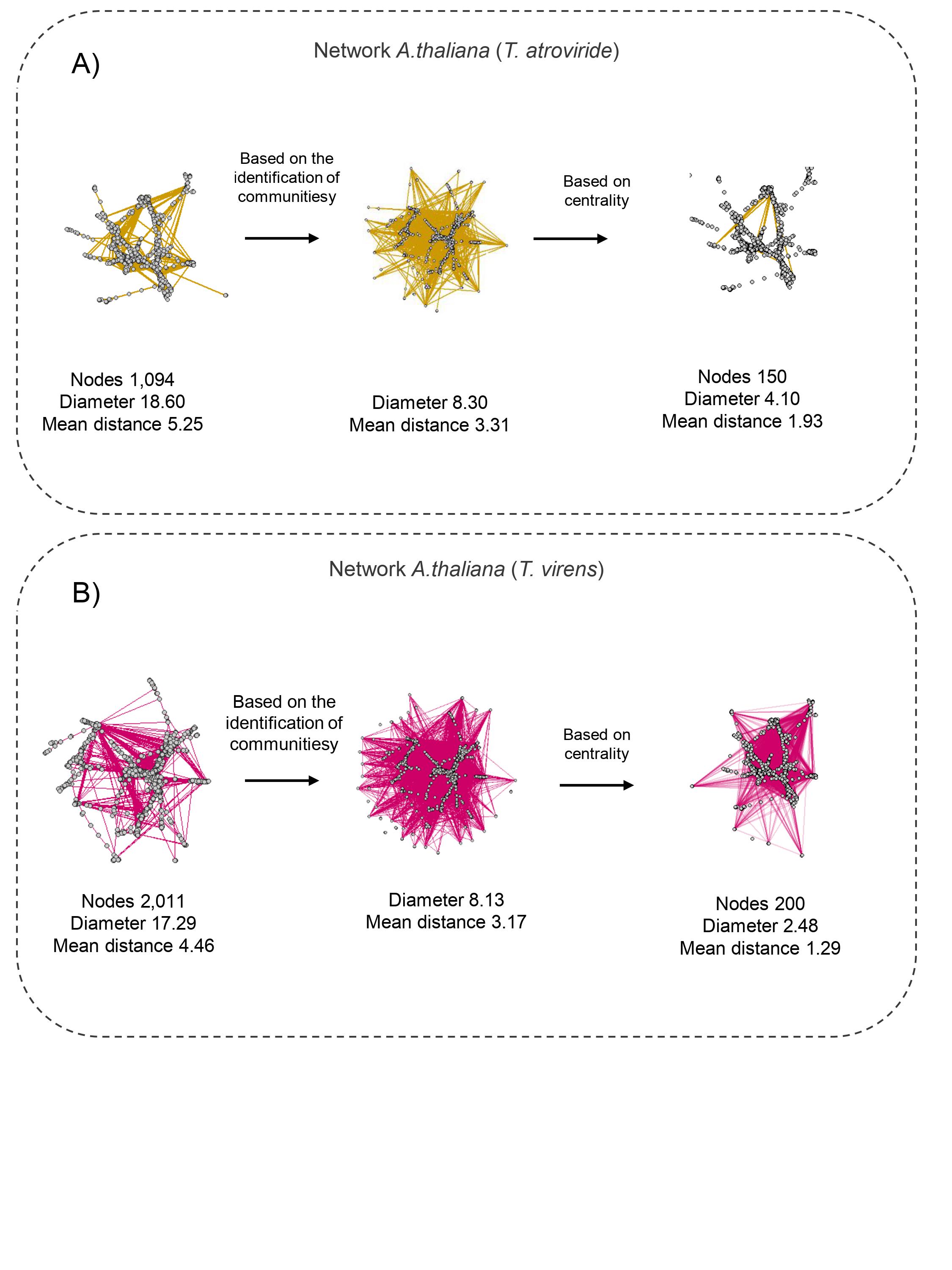


Figure S4. Generation and simplification of gene co-expression networks. A) *A. thaliana*(*T. atroviride*). A correlation-based network connecting genes with similar genetic interaction profiles. Genetic profile similarities were measured for all gene pairs by Spearman correlation. This network is made up of 1,094 nodes. Community 4, 22 and 24 *A.thaliana*(*T.atroviride*) were identified. A Subgraph was generated using the best 50 nodes according to our score (authority*closeness) for each community. B) *A.thaliana*(*T.virens*). A correlation-based network connecting genes with similar genetic associations profiles. Genetic profile similarities were measured for all gene pairs by Sperman correlation. This network is made up of 2,011 nodes. Community 5 and 6 *A.thaliana*(*T.virens*).


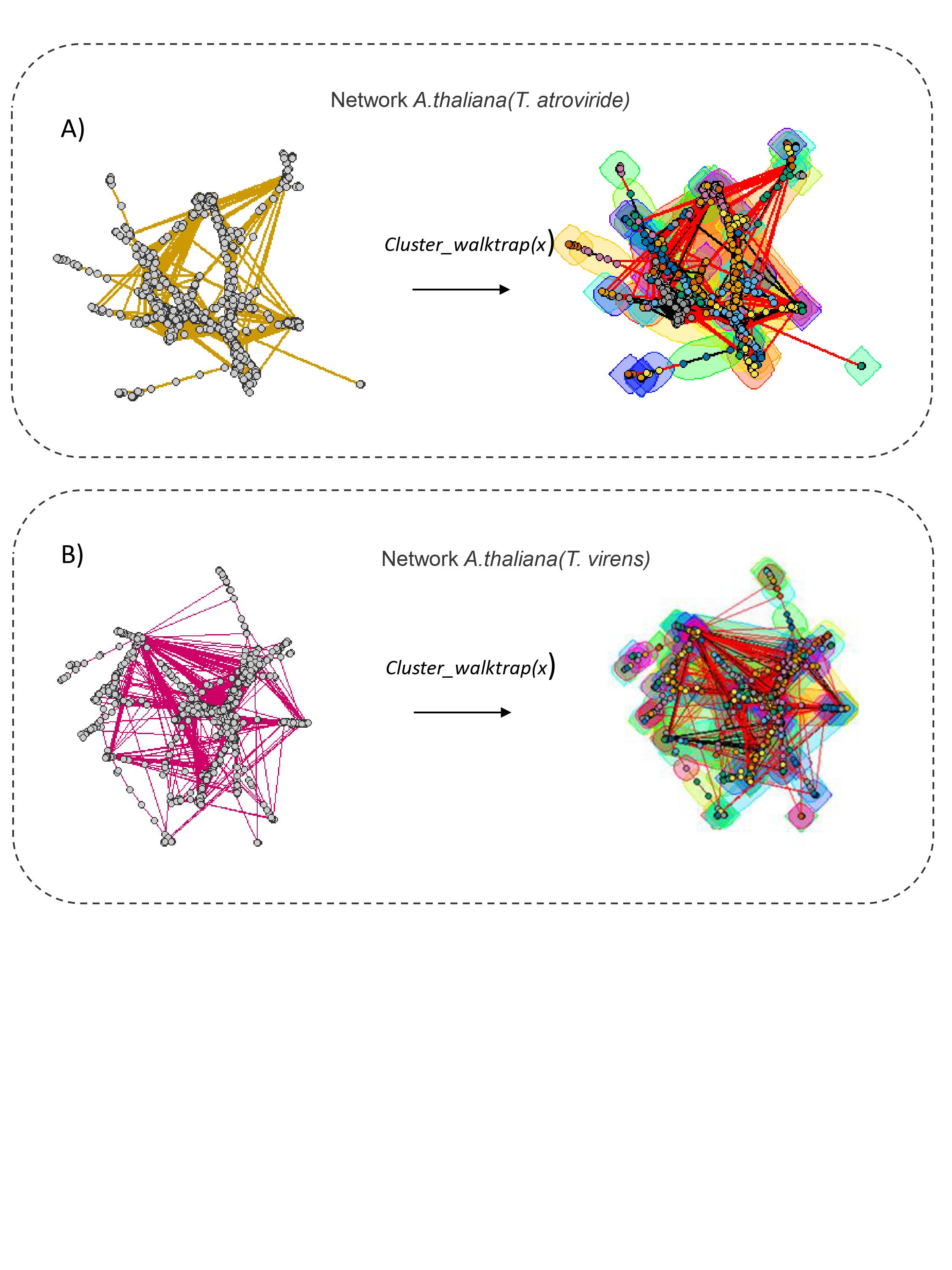


Figure S5. Identification of communities. A) *A.thaliana*(*T.atroviride*) and B) *A.thaliana(T.virens)*. The network shows the best 1,094 and 2,011 nodes for *A.thaliana*(*T.atroviride*) and *A.thaliana(T.virens) respectively*. The identification of communities was carried out using the function *cluster_walktrap(x)* of the igraph package. Each community is represented by a color. The analysis identified 104 communities for the A. thaliana(*T.atroviride*) network and 265 for the A.thaliana(*T.virens*) network


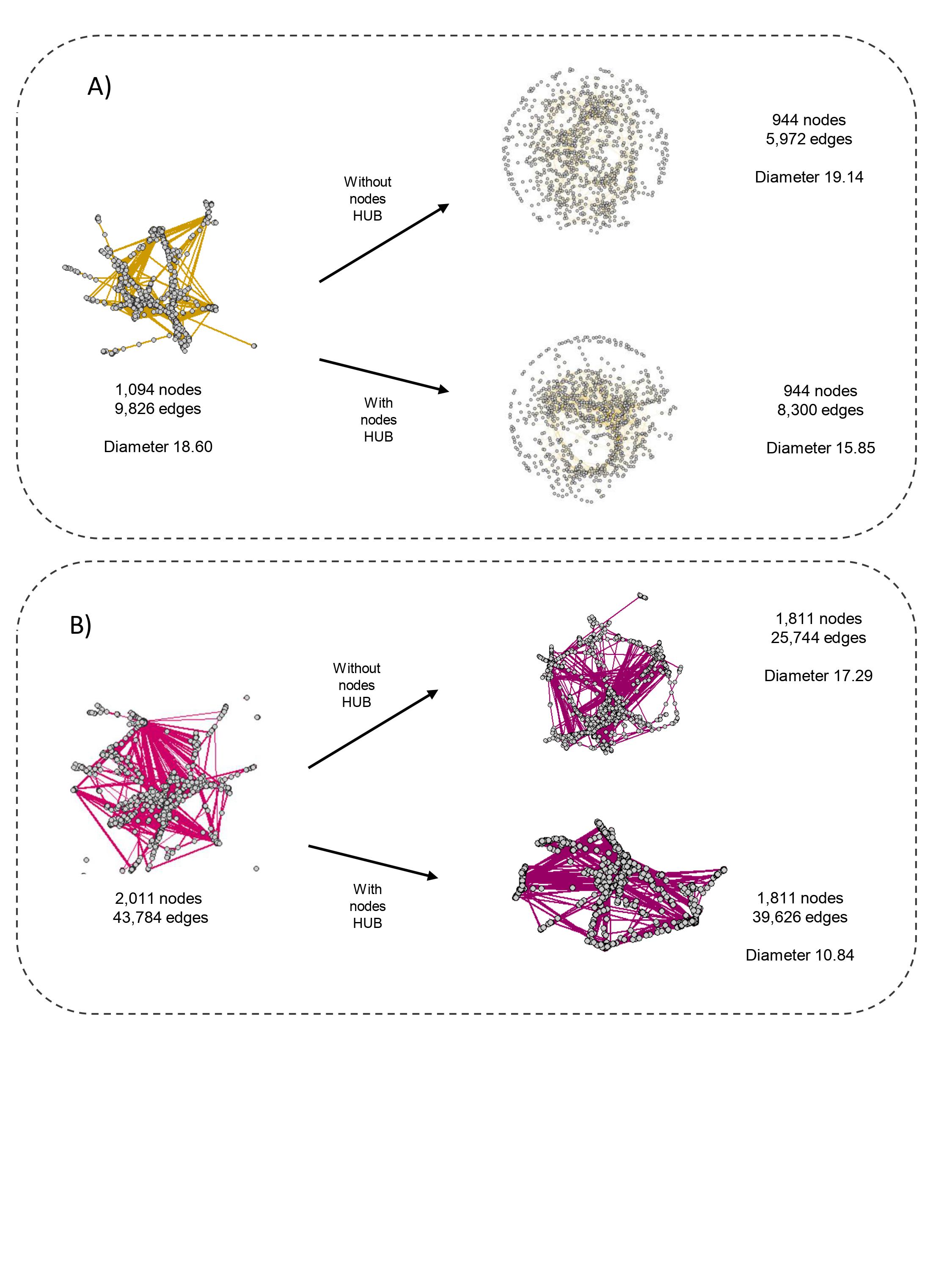


Figure S6. In silico validation of the selection of relevant nodes. A) Subgraph *A.thaliana*(*T.atroviride*). The best 150 nodes according to our score (authority x closeness x degree). In the first case the relevant nodes (the best score) were removed. In the second case nodes with bad score were removed. B) Subgraph *A.thaliana*(*T.virens*). The best 200 nodes according to our score (authority x closeness X degree). In the first case the relevant nodes (the best score) were removed. In the second case nodes with bad score were removed.


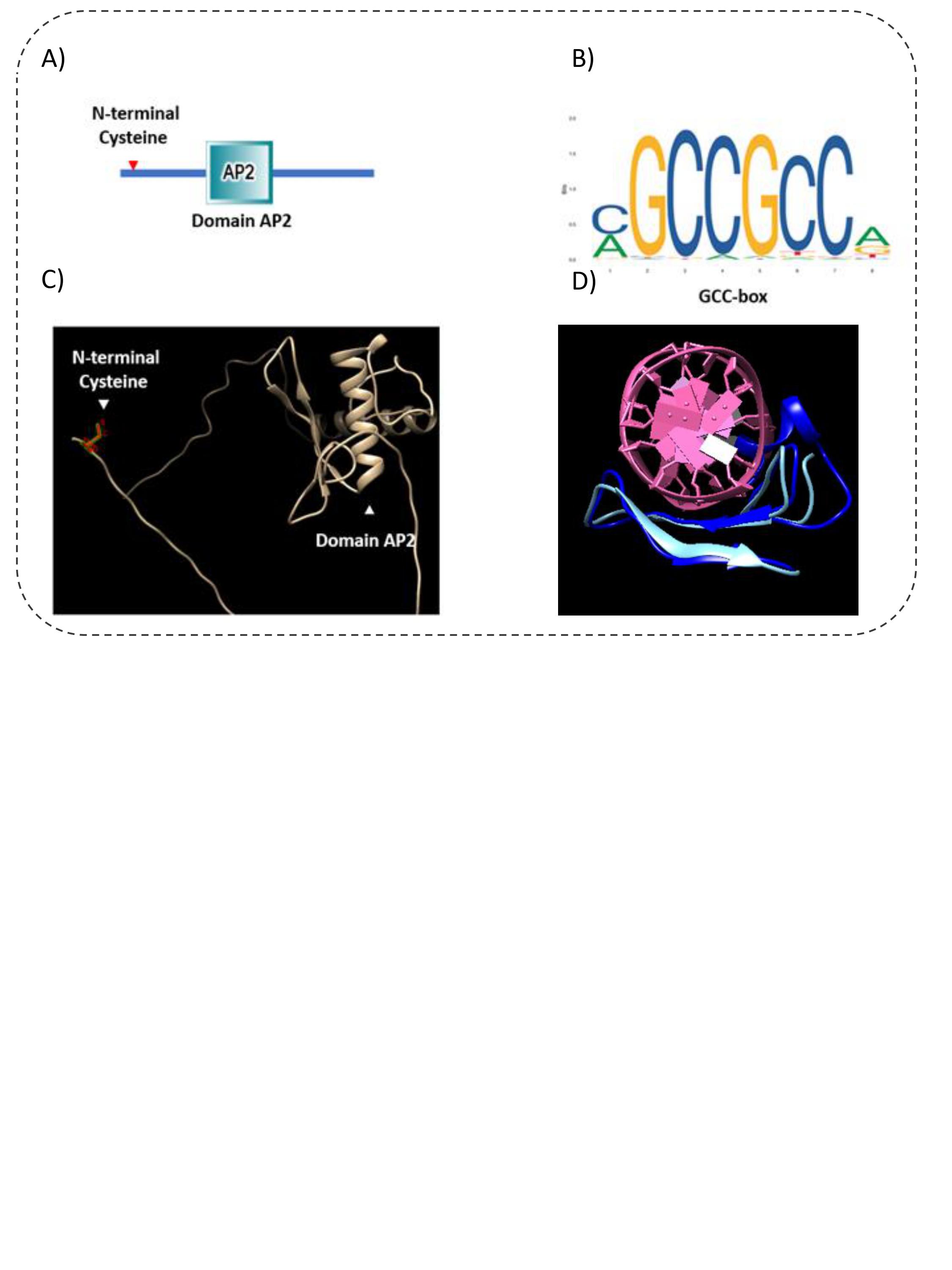


Figure S7. Identification of domains present in *AT2G47520* and analysis of position weight matrix (PWM). A) Domain identification. The analysis of conserved domains was carried out with the Conserved Domains Database (CDD). B) Analysis of position weight matrix (PWM). The analysis of positional weight matrices (PWM) with open-access database of curated JASPAR. Each letter and color represent a nucleotide of the GCC-box promoter sequence. The size of each letter represents the probability of binding between protein and DNA. C) Representation of domains in ribbon diagram. The structural prediction of the protein was performed using the alpha fold artificial intelligence program and was manipulated in CHIMERA. D) Structural alignment. Purple represents the GCC-box. Dark blue represents the binding domain to the GCC-box sequence of the ERF091 transcription factor. Light blue represents the binding domain to DNA of *AT2G47520*
